# *Notes from the Field: Acanthamoeba* Keratitis Cases — Iowa, 2002–2017

**DOI:** 10.15585/mmwr.mm6819a6

**Published:** 2019-05-17

**Authors:** Brittni A. Scruggs, Tyler S. Quist, Jorge L. Salinas, Mark A. Greiner

**Affiliations:** ^1^Department of Ophthalmology and Visual Sciences, Carver College of Medicine, University of Iowa, Iowa City; ^2^Division of Infectious Diseases, Department of Internal Medicine, Carver College of Medicine, University of Iowa, Iowa City; ^3^Iowa Lions Eye Bank, Coralville, Iowa.

*Acanthamoeba* is a ubiquitous protozoan that feeds on bacteria and yeast. Because of its ability to encyst in extreme environmental conditions, the organism is difficult to kill. Contact lens wearers exposed to any water source are at highest risk for developing *Acanthamoeba* keratitis (AK), a severe corneal infection that can result in painful blindness. Variable findings when patients seek treatment contribute to the underdiagnosis of AK, and the amoeba’s resistance to killing makes AK a challenging infection to treat for ophthalmologists. The incidence of *Acanthamoeba* keratitis in the United States is estimated to be one to two new cases per 1 million contact lens wearers annually (*1*); approximately 16.7% of U.S. adults wear contact lenses (*2*). Among the estimated 2.42 million Iowa residents aged ≥18 years, including an expected 404,267 adult contact lens wearers (16.7%), 0.4–0.8 new AK cases per year would be expected in Iowa. However, the University of Iowa Hospitals & Clinics (UIHC), the only tertiary care center in Iowa, diagnosed 15 new AK cases in 2015, 14 of which were adults. Because of this apparent excess in occurrent cases, a retrospective investigation was performed to ascertain AK cases evaluated at UIHC during a 16-year period.

Electronic records were queried to identify patients with corneal infections treated at UIHC during 2002–2017. A confirmed AK case was defined as detection of *Acanthamoeba* using confocal microscopy or corneal scraping, which was analyzed by an ocular pathologist for amoebic cysts. Overall, 111 confirmed AK cases were identified, including 75 (67.6%) in Iowa residents. For the following reasons, it is unlikely that any Iowa AK cases defined using the criteria described are not included in this cohort: AK cannot be diagnosed empirically, and UIHC uses the only clinical ophthalmic confocal microscope in Iowa and employs the only ocular pathologist in the state.

During 2002–2017, Iowa’s adult population increased 7.3%, from 2.25 million to 2.41 million persons*; 27,539 (16.7%) of the new 164,905 Iowa residents aged ≥18 years were expected to have been contact lens wearers. This increase in the population at risk for AK (i.e., one to two new cases per 1 million contact lens wearers annually) corresponds to an expected increase of 0.03–0.06 new AK cases in 2017, compared with 2002. However, among all Iowa residents, the average number of new AK cases per year increased from 2.9 cases during 2002–2009 to 6.5 cases during 2010–2017. The number of new AK cases doubled among residents during 2012–2014 (Figure).

**FIGURE:**
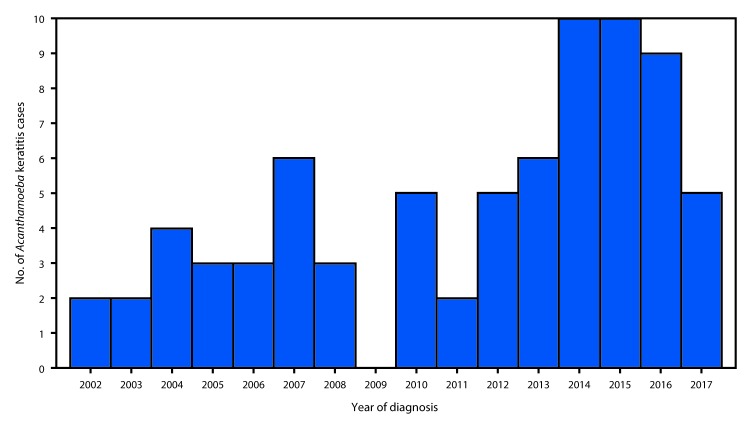
Confirmed cases* (N = 75) of *Acanthamoeba* keratitis diagnosed in Iowa residents, by year of diagnosis — University of Iowa Hospitals & Clinics, 2002–2017 * Detection of *Acanthamoeba* by confocal microscopy or corneal scraping among patients with corneal infection treated at University of Iowa Hospitals & Clinics during 2002–2017.

Among Iowans with confirmed AK, the median age was 32 years (range = 8–80 years), and 40 (53.3%) patients were women. Six (8%) patients were aged <18 years. Most patients had at least one risk factor, including contact lens wear (63; 84.0%), corneal injury (12; 16.0%), or eye exposure to organic material (e.g., dirt or algae) (12; 16.0%). Among the 63 affected contact lens wearers, 54 (85.7%) reported inadequate contact lens hygiene, and the majority reported recent water exposure, including swimming in lakes or rivers while wearing contacts (14; 22.2%), showering while wearing contacts (11; 17.5%), or cleaning contacts with tap water (eight; 12.7%). Twenty-one (33.3%) patients reported wearing contacts while sleeping, and 19 (30.2%) wore their contacts for longer than recommended by the manufacturer. AK was most prevalent in the summer (23 cases; 30.7%), followed by fall (21; 28.0%), winter (16; 21.3%), and spring (15; 20.0%). The average interval from symptom onset to diagnosis was 1.2 months (range = 0–4.4 months). Late diagnoses contributed to poor vision, and 31 Iowans with AK (41.3%) were legally blind (visual acuity <20/200) in the affected eye at first evaluation. Thirty-five (46.7%) patients ultimately required surgical intervention because of *Acanthamoeba* resistance to medical therapy.

AK is a sight-threatening condition, and outbreaks have been linked to specific contact lens solutions (*3*), hard water with lime scale (*4*), and biofilm contamination of domestic and recreational water (*5*,*6*). The expanding problem of algal bloom and nutrient pollution in Iowa waters might also be contributing to an increase in pathogenic *Acanthamoeba* species and the rising number of AK cases (*6*). Although this investigation was limited by its retrospective design and analysis of data obtained from a single center, UIHC is likely to evaluate most AK cases in Iowa. To prevent contact lens–related infections, including AK, CDC recommends that contact lens users not use tap water to clean contacts, swim or shower while wearing contacts, wear contacts for longer than recommended, or wear contacts while sleeping (*7*).

## References

[1] Schaumberg DA, Snow KK, Dana MR. The epidemic of *Acanthamoeba* keratitis: where do we stand? Cornea 1998;17:3–10. 10.1097/00003226-199801000-00001 9436873

[2] Cope JR, Collier SA, Rao MM, Contact lens wearer demographics and risk behaviors for contact lens-related eye infections—United States, 2014. MMWR Morb Mortal Wkly Rep 2015;64:865–70. 10.15585/mmwr.mm6432a2 26292204 PMC5779588

[3] Imayasu M, Tchedre KT, Cavanagh HD. Effects of multipurpose solutions on the viability and encystment of *Acanthamoeba* determined by flow cytometry. Eye Contact Lens 2013;39:228–33. 10.1097/ICL.0b013e31828af147 23584044

[4] Seal D, Stapleton F, Dart J. Possible environmental sources of *Acanthamoeba* spp in contact lens wearers. Br J Ophthalmol 1992;76:424–7. 10.1136/bjo.76.7.424 1627513 PMC504304

[5] Kilvington S, Gray T, Dart J, *Acanthamoeba* keratitis: the role of domestic tap water contamination in the United Kingdom. Invest Ophthalmol Vis Sci 2004;45:165–9. 10.1167/iovs.03-0559 14691169

[6] Abdul Majid MA, Mahboob T, Mong BG, Pathogenic waterborne free-living amoebae: an update from selected Southeast Asian countries. PLoS One 2017;12:e0169448. 10.1371/journal.pone.0169448 28212409 PMC5315373

[7] Cope JR, Konne NM, Jacobs DS, Corneal infections associated with sleeping in contact lenses—six cases, United States, 2016–2018. MMWR Morb Mortal Wkly Rep 2018;67:877–81. 10.15585/mmwr.mm6732a2 30114003 PMC6095652

